# Cluster Randomized Pragmatic Clinical Trial Testing Behavioral Economic Implementation Strategies to Improve Tobacco Treatment for Patients With Cancer Who Smoke

**DOI:** 10.1200/JCO.23.00355

**Published:** 2023-07-19

**Authors:** Brian P. Jenssen, Robert Schnoll, Rinad S. Beidas, Justin Bekelman, Anna-Marika Bauer, Sarah Evers-Casey, Tierney Fisher, Callie Scott, Jody Nicoloso, Peter Gabriel, David A. Asch, Alison M. Buttenheim, Jessica Chen, Julissa Melo, Dwayne Grant, Michael Horst, Randall Oyer, Lawrence N. Shulman, Alicia B.W. Clifton, Adina Lieberman, Tasnim Salam, Katharine A. Rendle, Krisda H. Chaiyachati, Rachel C. Shelton, Oluwadamilola Fayanju, E. Paul Wileyto, Sue Ware, Daniel Blumenthal, Daniel Ragusano, Frank T. Leone

**Affiliations:** ^1^ Department of Pediatrics, Perelman School of Medicine, University of Pennsylvania, Philadelphia, PA; ^2^ Department of Psychiatry, Perelman School of Medicine, University of Pennsylvania, Philadelphia, PA; ^3^ Abramson Cancer Center, Perelman School of Medicine, University of Pennsylvania, Philadelphia, PA; ^4^ Perelman School of Medicine, University of Pennsylvania, Philadelphia, PA; ^5^ Department of Medical Social Sciences, Feinberg School of Medicine, Northwestern University, Chicago, IL; ^6^ Department of Radiation Oncology, Hospital of the University of Pennsylvania, Philadelphia, PA; ^7^ Comprehensive Smoking Treatment Program, Perelman School of Medicine, University of Pennsylvania, Philadelphia, PA; ^8^ Department of Family and Community Health, School of Nursing, University of Pennsylvania, Philadelphia, PA; ^9^ Penn Medicine Lancaster General Health, Lancaster, PA; ^10^ Department of Family Medicine and Community Health, Perelman School of Medicine, University of Pennsylvania, Philadelphia, PA; ^11^ Center for Clinical Epidemiology and Biostatistics, Perelman School of Medicine, University of Pennsylvania, Philadelphia, PA; ^12^ Verily Life Sciences, San Francisco, CA; ^13^ Department of Sociomedical Sciences, Mailman School of Public Health, Columbia University, New York, NY; ^14^ Department of Surgery, Perelman School of Medicine, University of Pennsylvania, Philadelphia, PA; ^15^ Pulmonary, Allergy, & Critical Care, Perelman School of Medicine, University of Pennsylvania, Philadelphia, PA

## Abstract

**PURPOSE:**

Few cancer centers systematically engage patients with evidence-based tobacco treatment despite its positive effect on quality of life and survival. Implementation strategies directed at patients, clinicians, or both may increase tobacco use treatment (TUT) within oncology.

**METHODS:**

We conducted a four-arm cluster-randomized pragmatic trial across 11 clinical sites comparing the effect of strategies informed by behavioral economics on TUT engagement during oncology encounters with cancer patients. We delivered electronic health record (EHR)–based nudges promoting TUT across four nudge conditions: patient only, clinician only, patient and clinician, or usual care. Nudges were designed to counteract cognitive biases that reduce TUT engagement. The primary outcome was TUT penetration, defined as the proportion of patients with documented TUT referral or a medication prescription in the EHR. Generalized estimating equations were used to estimate the parameters of a linear model.

**RESULTS:**

From June 2021 to July 2022, we randomly assigned 246 clinicians in 95 clusters, and collected TUT penetration data from their encounters with 2,146 eligible patients who smoke receiving oncologic care. Intent-to-treat (ITT) analysis showed that the clinician nudge led to a significant increase in TUT penetration versus usual care (35.6% *v* 13.5%; OR = 3.64; 95% CI, 2.52 to 5.24; *P* < .0001). Completer-only analysis (N = 1,795) showed similar impact (37.7% clinician nudge *v* 13.5% usual care; OR = 3.77; 95% CI, 2.73 to 5.19; *P* < .0001). Clinician type affected TUT penetration, with physicians less likely to provide TUT than advanced practice providers (ITT OR = 0.67; 95% CI, 0.51 to 0.88; *P* = .004).

**CONCLUSION:**

EHR nudges, informed by behavioral economics and aimed at oncology clinicians, appear to substantially increase TUT penetration. Adding patient nudges to the implementation strategy did not affect TUT penetration rates.

## INTRODUCTION

Continued tobacco smoking by patients with cancer worsens quality of life (QoL) and reduces survival.^1-3^ Smoking accelerates tumor growth, disease progression, tumor resistance to treatment, and treatment-related toxicities.^4-7^ Yet, >50% of patients with cancer who smoked before their diagnosis continue to smoke after diagnosis and during treatment.^8^ Routine evidence-based tobacco use treatment (TUT) reduces cancer-specific and all-cause mortality, reduces treatment-related toxicity, and improves QoL among patients receiving cancer care.^1^ Therefore, the 2021 US Preventive Services Task Force, Healthy People 2030, the Department of Health and Human Services, and the Surgeon General have recommended that clinicians ask all adults about tobacco use, advise them to quit, and provide evidence-based treatment.^9-12^ Likewise, the National Comprehensive Cancer Network,^3^ American Society of Clinical Oncology,^13^ and the American Association for Cancer Research^14^ encourage oncologists to provide evidence-based TUT.

**CONTEXT**

**Key Objective**
Are nudges informed by behavioral economics during oncology encounters with patients with cancer effective for increasing engagement in tobacco use treatment (TUT)?
**Knowledge Generated**
Clinician nudges aimed at counteracting omission bias and delivered via the electronic health record resulted in a significant (>3-fold) increase in TUT engagement. The addition of a patient nudge did not affect TUTS engagement rates. Among clinicians, advanced practice providers were more likely than physicians to engage in TUT. White and non-White patients were equally engaged in TUT by the clinician nudge.
**Relevance *(S.B. Wheeler)***
Clinician-focused nudges can provide important and timely cues to action that result in more patients engaging in TUT. Such nudges may be a low-cost, low-risk way to optimize use of tobacco cessation services during clinical encounters.*
  *Relevance section written by *JCO* Associate Editor Stephanie B. Wheeler, PhD, MPH.

Despite the importance of TUT, only half of the cancer centers consistently identify patient tobacco use,^15^ and fewer systematically engage patients in evidence-based TUT.^16^ In response to this practice gap, the National Cancer Institute launched the Cancer Center Cessation Initiative (C3i) to help centers develop ways to identify and engage patients who smoke.^17^ One major C3i objective is overcoming clinician, patient, and health system barriers by integrating TUT into cancer care. An earlier C3i implementation strategy studied at Penn Medicine focused on maximizing tobacco use screening and referral, on the basis of the *Ask-Advise-Connect* model.^18^ Because lack of clinician experience is a frequently cited barrier to TUT,^19-21,^ our initial strategy used an automated default electronic health record (EHR) referral to our TUT Service (TUTS). This effort significantly improved TUT engagement, with TUTS referrals rising from 0% at baseline to 34% during the 6-month postimplementation period. Although this suggested that clinician behavior was modifiable, the study was not a randomized trial and >60% of default referral orders were declined, implicating additional implementation barriers.^22^

Implementation efforts to promote TUT engagement within oncology may be enhanced using behavioral economics, which has helped improve patient outcomes and transform health care delivery across a wide range of activities.^23-26^ Specific clinician-related barriers likely decrease TUT engagement, including clinician pessimism regarding the ability to help patients stop using tobacco, misconceptions about patient resistance, and implicit biases regarding patients’ capacities to volitionally alter the course of illness.^27^ These motivators are related to clinician willingness to invest effort in help-giving^28-30^ and may prevent acquisition of new skills.^31^ Individuals with cancer face unique challenges when engaging in tobacco cessation efforts, including low self-efficacy, low perceived benefits of quitting, and perceived treatment risk.^32-34^ Furthermore, racial and ethnic minorities and individuals with low socioeconomic status (SES) suffer disproportionately from targeted tobacco marketing, have diminished access to evidence-based TUT, and report poorer response to TUT.^35-39^ Thus, social inequities may also affect implementation and TUT engagement.

We designed this pragmatic trial to test the effectiveness of patient- and clinician-directed implementation strategies, or nudges, informed by behavioral economics and delivered through the EHR, to counteract heuristics that reduce the likelihood of engaging in TUT. We compared the effects of nudges directed at patients, clinicians, or both to usual care on rates of TUT. We also performed a preliminary examination of patient and clinician characteristics that may moderate the impact of nudges on rates of TUT, including characteristics that may have important implications for health and social inequities, such as patient age, sex, race/ethnicity, and neighborhood-level SES.

## METHODS

### Design and Setting

We conducted a cluster-randomized pragmatic trial across five hospitals and six clinics within Penn Medicine's Abramson Cancer Center.^40^ Clinician clusters were randomized into four arms: clinician nudge, patient nudge, both clinician and patient nudge, or usual care. Patients were nested within clinician clusters. The primary outcome was *penetration* of TUT, defined as the provision of a patient referral for the TUTS program or the provision of tobacco use medication during cancer care. This study was approved by the University of Pennsylvania Institutional Review Board. Since this was a pragmatic trial to improve use of evidence-based tobacco treatments, the study represented minimal participant risk, and a waiver of informed consent was granted.

### Participant Eligibility

The clinician sample included physicians and advanced practice providers (APPs) within medical, radiation, and gynecologic oncology clinics. Eligibility criteria for clinicians included (1) currently practicing at an included site; (2) prescribing authority in Pennsylvania or New Jersey; (3) cared for ≥1 patient who used tobacco within the 30-day period preceding recruitment; and (4) English-speaking. Eligibility criteria for patients included any International Classification of Diseases-10 cancer diagnosis, self-reported current tobacco use assessed by staff initiating the visit, a scheduled appointment with a participating clinician, and English-speaking. Patients were accrued as they were seen by an eligible clinician.

### Study Procedures

**FIG 1.**
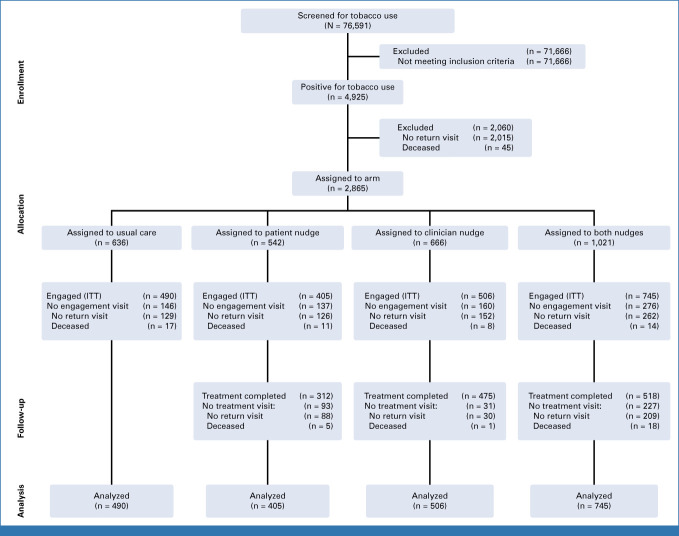
CONSORT diagram. For the assigned to usual care arm, treatment visit = 0 because of the nature of the intervention design. There are no nudges to be delivered. In other words, treatment is completed with the engaged visit, and there are no additional treatment visits. ITT, intent-to-treat.

**FIG 2.**
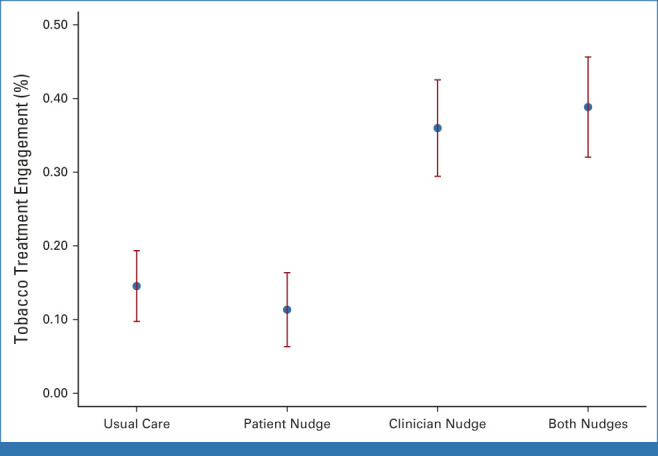
Rates of tobacco treatment engagement across treatment arms (ITT model). ITT, intent-to-treat.

Clinician enrollment proceeded in two steps: (1) announcement of study initiation at staff meetings, with opportunity to ask questions about design and impact on workflow, and (2) a personalized email delivered to all eligible clinicians reiterating study methods and providing instructions for opting out. All eligible clinicians (N = 246) were enrolled, with none opting out when offered. Patient enrollment began with a positive tobacco use assessment at the first patient visit within the study period, termed the *index visit.* Assessment of patient tobacco exposure included the 30-day period preceding the index visit, and was accomplished using a standardized Best Practice Alert (BPA) activated within the EHR during the check-in and vital sign workflow.^22^ The next scheduled visit with a clinician in a cluster randomly assigned to that same arm was termed the *subsequent visit*, during which clinicians received a care guidance BPA at the point of care if randomly assigned to the clinician nudge or the clinician and patient nudge (Data Supplement [Figs 1 and 2], online only). Clinicians randomly assigned to the patient nudge arm received no care guidance BPA. Instead, their patients received an electronic message 24-72 hours before the subsequent visit encouraging them to speak with the clinician about TUT. Patient nudges were delivered through *myPennMedicine*, the patient portal used by >75% of patients with cancer. If randomly assigned to the both-nudges arm, both patients and clinicians received nudges as above. Usual care subsequent visits proceeded without either nudge.

### Intervention Content

#### Clinician Nudge

The findings from our preliminary work examining physician preferences toward TUT revealed a strong preference for interventions perceived as effective.^41^ We previously showed that strategies minimizing well-established cognitive biases such as omission bias—the tendency to focus on the potential harm of action more than that of inaction—change physician behavior more than strategies solely aiming to increase knowledge of TUTS availability.^42^ We developed a BPA that targeted this bias by directly reminding clinicians that “[t]reating tobacco dependence without delay can improve cancer care outcomes” and facilitating an instant referral to TUTS (Data Supplement [Figs 1 and 2]). Clinicians were required to either accept the defaulted send option, or to deselect the command and actively choose the do not send option. Clinicians opting out were required to provide a justification using an available checklist or free text. The BPA did not restrict clinicians' ability to directly provide TUT by either prescribing medication or referring for TUT through alternative mechanisms.

#### Patient Nudge

Status quo bias, or sticking with a current choice even if better alternatives exist, can reduce patient willingness to engage in TUT.^43^ The patient nudge included information specific to an upcoming appointment with the oncology clinician and encouraged patients to discuss TUT with their clinician by emphasizing the importance of TUT to their care, delivered via the online portal (Data Supplement [Figs 1 and 2]).

#### Usual Care

Clinicians can refer to TUTS or offer TUT on their own without prompting. Our previous evidence suggests this rarely, if ever, happens.^22^

### Measures

During preparation for this trial, we identified alternate workflows that clinicians used to provide tobacco use medications without referring patients to the TUT program.^40^ We chose an inclusive, pragmatic outcome definition of penetration to capture all observable TUT behaviors. Thus, the primary implementation outcome was penetration of TUT, defined as the proportion of patients who received either a treatment referral (via the BPA or elsewhere in the EHR workflow) or a prescription for tobacco treatment medication (ie, nicotine replacement, varenicline, or bupropion). We collected patient-level data, including age, sex, and race/ethnicity, from the EHR. We also calculated neighborhood-level SES at the census tract level using the Yost score, a validated composite index of SES incorporating variables such as education, income, and occupation.^44^ Clinician-level data included sex, race, clinician type (physician *v* APP), and specialty. Practice-level data included geographic location.

### Random Assignment

We randomized by clinician clusters identified on the basis of paired connections between physicians and APPs within networks of practice colleagues. Clusters were formed between clinicians with overlapping patient pools to reduce cross-cluster contamination. The clusters were not site-specific as many clinicians worked at multiple sites. We formed 95 clusters from 246 clinicians. Patients were nested under clinician clusters and assigned to an arm based upon the clinician they saw at their index visit, preventing crossover.

### Statistical Analysis

A sample of 900 patients provided ≥80% power to detect an 11% improvement in our primary outcome (eg, from 34% referral rate for current estimates to a clinically relevant 45%), using a two-sided type 1 error rate of 5% and an interclass correlation of 0.07, for planned comparisons between usual care and each nudge arm. We analyzed our binary outcome using logistic regression with generalized estimating equations (GEE). The study design is factorial, and models contained binary predictor terms for clinician and/or patient nudges. We included covariates from patients (eg, race) and clinicians (eg, clinician type) and controlled for time between visits in days. We controlled for type 1 error inflation by hierarchical testing, starting with the overall model significance, followed by effects of each nudge. Once we fitted the main effects model, we tested for interaction between nudges and retained the interaction term if significant. We followed the primary modeling with post hoc assessments of predictors, including our a priori interest in race, to assess interactions with study arm. Our primary analysis was intent-to-treat (ITT) so that all patients who completed the subsequent visit were included regardless of whether all interventions were received (N = 2,146). In a secondary analysis, we examined a GEE model that included only encounters wherein all nudges were received as intended (ie, a completer-only analysis; N = 1,795).

## RESULTS

### Sample Characteristics and Covariates

Figure 1 shows the accrual and randomization data from this trial. Seventy-six thousand five hundred ninety-one patients were screened for current tobacco use between June 2021 and July 2022. Of the 4,925 (6.4%) patients who screened positive for tobacco use, 2,865 (58%) had a scheduled visit with a clinician enrolled in this study; 2,146 patients (75%) completed a subsequent clinic visit and formed the ITT sample. Average time between index and subsequent visits was 70.1 days (standard deviation = 71.4). However, accelerated visit schedules prevented some in the ITT sample from receiving nudges before their subsequent visit (n = 351), yielding a completer-only sample of 1,795. Tables 1 and 2 show the sample characteristics.

**TABLE 1.**
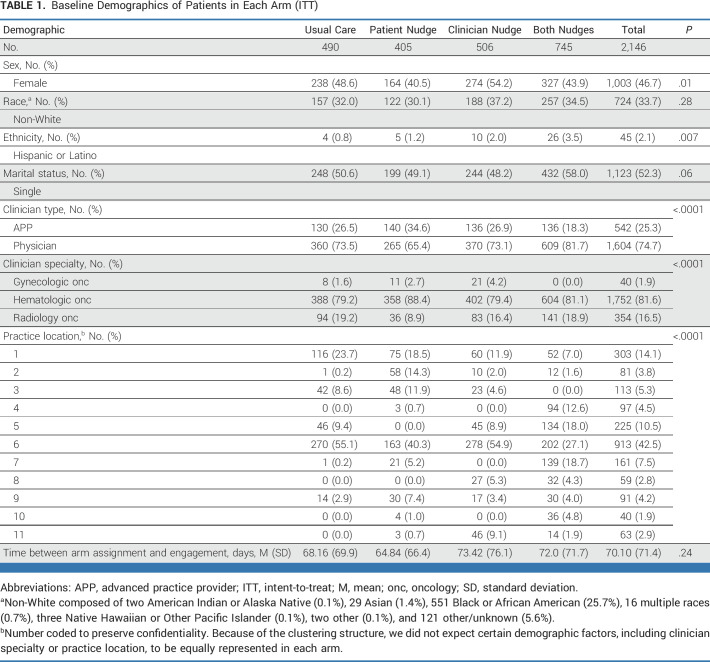
Baseline Demographics of Patients in Each Arm (ITT)

**TABLE 2.**
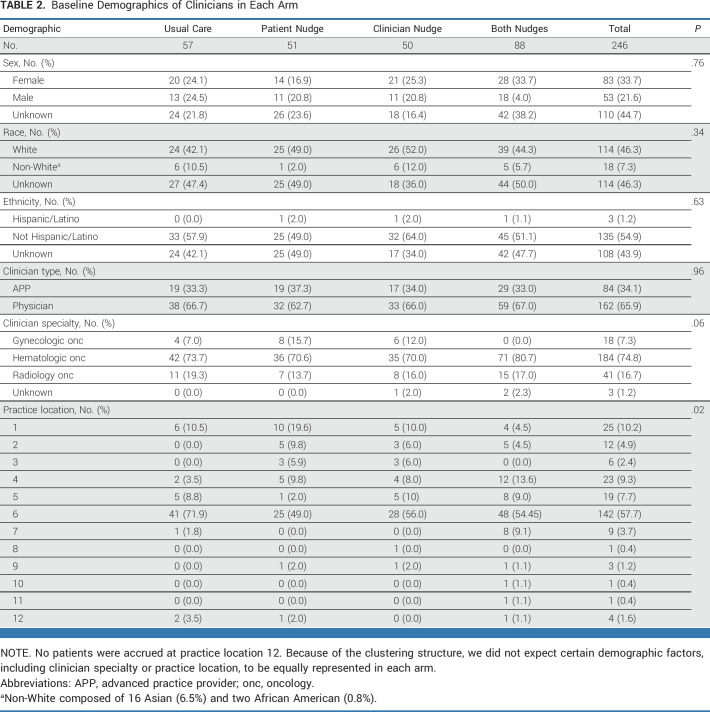
Baseline Demographics of Clinicians in Each Arm

For patients who received the patient nudge, 55% opened the message, and, of those, 85% opened it on the day it was sent (average time to open = 1.39 days). Patients who did not receive the nudge were older (62.9 *v* 62.2; F[1,2144] = 5.03; *P* = .02), and more likely to be male (17.8% *v* 14.7%; χ^2^[1] = 3.997; *P* = .047), non-White (20.2% *v* 14.4%; χ^2^[1] = 11.3; *P* = .001), Hispanic (33.3% *v* 16%; χ^2^[1] = 7.95; *P* = .004), single (18.7% *v* 13.8%; χ^2^[1] = 9.53; *P* = .002), or seen by a gynecologic oncologist (30% *v* 15.5% *v* 18.9%; χ^2^[2] = 7.13; *P* = .03).

### Models of TUT

All models controlled for variables in Tables 1 and 2 that were different across arms except for location in the ITT model since that variable was missing for participants for whom the nudge was not delivered. Controlling for covariates, both the ITT and completer-only models predicting TUT penetration were significant (ITT: χ^2^[12] = 115.94; *P* < .0001; completers: χ^2^[11] = 130.46; *P* < .0001). Although neither model showed significant interaction effects for the clinician and patient nudges, the clinician nudge arm main effect was a significant predictor of TUT penetration (Table 3). Compared with usual care, the clinician nudge yielded about a 3-fold increase in rates of penetration of TUT (Fig 2 and completer—Table 4).

**TABLE 3.**
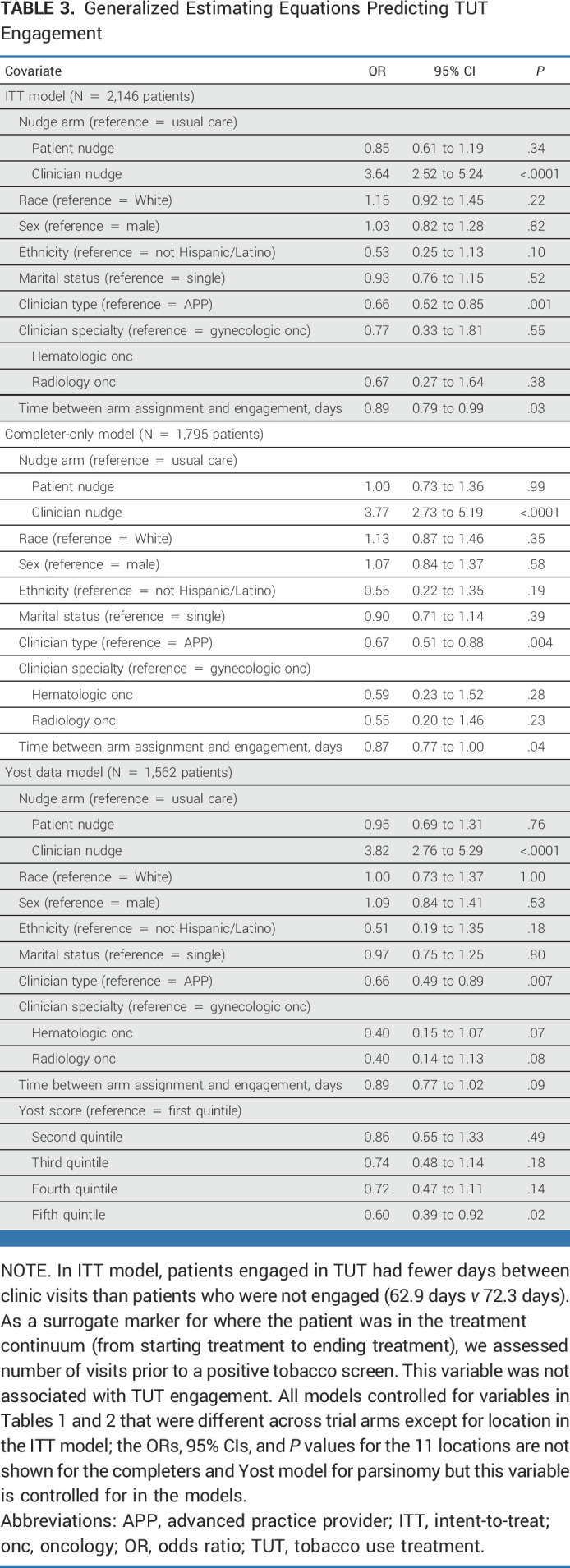
Generalized Estimating Equations Predicting TUT Engagement

**TABLE 4.**
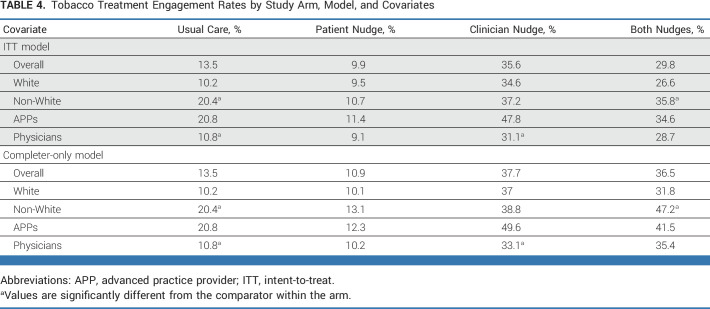
Tobacco Treatment Engagement Rates by Study Arm, Model, and Covariates

In both models, clinician type was associated with TUT penetration rates (Table 3). There was no difference between APPs and physicians within the patient-only and combined nudge arms (Table 4), but higher penetration rates were found for APPs in the usual care (χ^2^[1] = 7.51; *P* = .007) and the clinician-only (χ^2^[1] = 11.85; *P* = .001) arms. Likewise, in the completer-only model, patients seen by APPs experienced higher TUT rates in the usual care (χ^2^[1] = 7.51; *P* = .007) and the clinician nudge (χ^2^[1] = 10.78; *P* = .001) arms.

Race was not associated with penetration of TUT in the models (Table 3). However, on the basis of our a priori interest in assessing equity, our post hoc analyses suggested an uneven impact of race across arms in the completers model (Table 4): non-White patients had significantly higher TUT rates in the usual care (χ^2^[1] = 8.97; *P* = .003) and both-nudges (χ^2^[1] = 11.12; *P* = .01) arms. This pattern of TUT penetration across arms and race was similar in the ITT model: higher TUT rates were observed in the usual care (χ^2^[1] = 8.97; *P* = .003) and both-nudges (χ^2^[1] = 6.65; *P* = .011) arms, but not in the patient or clinician nudge arms, suggesting that the influence of race on the completer-only model may have been a proxy for the influence of race across all arms. Restricting the completer-only model to include only the subset of patients for whom geocoding data were available through the EHR (n = 1,562) and adding Yost score as a covariate did not affect the main effects and showed that TUT penetration was lower in the highest SES group (Table 4).

## DISCUSSION

Nudges delivered through the EHR are a well-established way to affect clinical behaviors and can have a substantial effect on patient and implementation outcomes.^45^ Changes to the way information is offered at the point of decision making are considered ethically acceptable if opting out of the default option remains easy and the range of clinical choices is preserved.^46^ In previous examples, clinician nudges using behavioral economic principles helped increase adherence to targeted guidelines, including for statin initiation,^47^ vaccination,^48^ and lung cancer screening.^49^ To our knowledge, this is the first study in oncology to compare implementation strategies to improve TUT using behavioral economic nudges aimed at clinicians, patients, or both. It builds upon our previous work and targets theoretically informed biases among both clinicians and patients, addressing known barriers to tobacco cessation treatment in this high-risk population. Overall, our results indicate that clinician nudges, aimed at counteracting omission bias and using default options and accountable justification of deviation, significantly increase the odds of TUT during cancer care—an effect that can benefit patients in both the short and long term.^50^

The impact of patient-directed nudges is less well established, and depends on the style of the nudge and the nature of the target problem.^51,52^ When considering nudging tobacco dependence treatment in cancer care, a shift in pre-existing assumptions is clearly warranted.^53^ Because smoking is stigmatized, it remained unclear what impact a patient-directed nudge might have on the clinical interaction. A nudge perceived as overly directive or judgmental might discourage engagement during the clinic visit or, in the worst case, undermine therapeutic relationships.^54^ Our patient nudge attempted to frame TUT positively and counteract the influence of status quo bias. Although our clinician nudge used techniques higher on the ladder of nudge interventions, our patient nudge used a lower-potency approach.^55^ Our inability to identify a discernable effect of the patient nudge may be a function of potency, characteristics of the electronic message transmission vehicle, or the specific cognitive bias addressed. Race was not a significant predictor of TUT penetration in the models but our post hoc assessment of this relationship indicated race could be a factor in TUT penetration. This observation was difficult to explain but may have been due to clustering across a geographically large and diverse health system. Our observations on race may have been confounded by SES as well, with a significant inverse relationship between SES quintile and TUT penetration. In either case, nudges did not exacerbate traditional inequities that typically affect health care delivery.

When nudges are introduced to clinician workflows, they are particularly effective for increasing TUT penetration when APPs are handling clinical encounters. This may be due to training or perspective, with APPs engaging in this element of clinical care more readily than their physician counterparts. It is also likely that the details of cancer care workflow confound this association; physicians may be more likely to perform initial evaluations while APPs are more likely to perform follow-up care. If so, the over-representation of TUT among APPs may be a function of pragmatic care planning concerns.

There are key strengths and limitations of this work. We used a pragmatic design and engaged both patients and clinicians—a key strength.^56^ There is potential for these implementation strategies to be both highly impactful and generalizable to other clinical settings and systems. Because our outcomes focused on clinical behaviors that are generally the result of a negotiated plan between clinician and patient, one key limitation is that clinician decision making may be moderated by unmeasured patient refusals. Also, given the multidisciplinary nature of cancer care, the potential for confounding because of contamination was present despite our a priori efforts to minimize its effect (eg, penetration influenced by frequency and/or variety of clinical visits). Finally, about 17% of patient nudges were not effectively delivered and more remained unread. Patients who did not receive the nudge may have had more advanced stages of disease necessitating accelerated visit schedules, no online portal account created, or another health care access–related disparity. This represents a significant opportunity for enhancement. Nevertheless, the results were remarkably consistent across the ITT and completer-only models.

Overall, this well-powered, rigorous pragmatic study demonstrates that implementation strategies, informed by behavioral economics within the EHR to counter biases that reduce health behavior engagement, can significantly increase the penetration of TUT in oncology care and do so without exacerbating health inequities in care delivery. Future work is needed to test the ability to generalize these findings to other health systems and to optimize the patient-directed nudge. Such work can continue to demonstrate the impact of implementation science and behavioral economics for enhancing the quality of cancer care to promote the best possible clinical outcomes for patients.

## Data Availability

Participant data reported in the article, after deidentification, will be available at the time of publication, along with a data dictionary. A methodologically rigorous proposal to use the data should be provided to the corresponding author, Brian Jenssen (JenssenB@chop.edu). Data will be made available with limited investigator support after investigator approval of the proposal and approval by the University of Pennsylvania Perelman School of Medicine.
